# The prognostic value of derived neutrophil to lymphocyte ratio in oesophageal cancer treated with definitive chemoradiotherapy

**DOI:** 10.1016/j.radonc.2017.08.023

**Published:** 2017-10

**Authors:** Samantha Cox, Christopher Hurt, Tal Grenader, Somnath Mukherjee, John Bridgewater, Thomas Crosby

**Affiliations:** a Department of Oncology, Velindre Cancer Centre, Cardiff, United Kingdom; b Wales Cancer Trials Unit, Cardiff University, Cardiff, United Kingdom; c Oncology Institute, Shaare Zedek Medical Centre, Jerusalem, Israel; d Department of Oncology, Oxford Cancer Centre, University of Oxford, Churchill Hospital, Oxford, United Kingdom; e University College Hospital Macmillan Cancer Centre, London, United Kingdom

**Keywords:** Oesophageal cancer, Definitive chemoradiotherapy (dCRT), Derived neutrophil–lymphocyte ratio (dNLR), Prognostic biomarker

## Abstract

**Background and purpose:**

The derived neutrophil–lymphocyte ratio (dNLR) is a validated prognostic biomarker for cancer survival but has not been extensively studied in locally-advanced oesophageal cancer treated with definitive chemoradiotherapy (dCRT). We aimed to identify the prognostic value of dNLR in patients recruited to the SCOPE1 trial.

**Materials and methods:**

258 patients were randomised to receive dCRT ± cetuximab. Kaplan–Meier’s curves and both univariable and multivariable Cox regression models were calculated for overall survival (OS), progression free survival (PFS), local PFS inside the radiation volume (LPFSi), local PFS outside the radiation volume (LPFSo), and distant PFS (DPFS).

**Results:**

An elevated pre-treatment dNLR ≥ 2 was significantly associated with decreased OS in univariable (HR 1.74 [95% CI 1.29–2.35], *p* < 0.001) and multivariable analyses (HR 1.64 [1.17–2.29], *p* = 0.004). Median OS was 36 months (95% CI 27.8–42.4) if dNLR < 2 and 18.4 months (95% CI 14.1–24.9) if dNLR ≥ 2. All measures of PFS were also significantly reduced with an elevated dNLR. dNLR was prognostic for OS in cases of squamous cell carcinoma with a non-significant trend for adenocarcinoma/undifferentiated tumours.

**Conclusions:**

An elevated pre-treatment dNLR may be an independent prognostic biomarker for OS and PFS in oesophageal cancer patients treated with definitive CRT. dNLR is a simple, inexpensive and readily available tool for risk-stratification and should be considered for use in future oesophageal cancer clinical trials.

The SCOPE1 trial was an International Standard Randomised Controlled Trial [number 47718479].

Oesophageal cancer is the 13th most common cancer in the UK with approximately 8800 new diagnoses each year [1]. Despite steady improvements in treatment outcomes over the last four decades, the majority of patients present with advanced disease and 5-year survival rates remain low at round 15% [1].

The SCOPE1 (Study of Chemoradiotherapy in OesoPhageal Cancer with or without Erbitux) trial has standardised radiotherapy treatment protocols within the UK [2]. The trial was closed at the phase II stage due to higher rates of toxicity and poorer survival outcomes in patients randomised to CRT with cetuximab [3]. However the long-term outcomes have demonstrated survival rates similar to surgical studies, with a median overall survival of 34.5 months (95% CI 24.7–42.3 months) for patients treated with cisplatin/capecitabine-based CRT [4]. This has added to the growing evidence that definitive chemoradiotherapy (dCRT) is a comparable curative treatment option in selected patient groups, particularly in cases of locally advanced squamous cell carcinoma or if surgery is unfeasible due to extent of disease or patient co-morbidities [5], [6], [7].

In an era of personalised medicine, the use of robust prognostic factors is being investigated to further improve outcomes as risk-stratification at the point of diagnosis could allow an appropriate treatment strategy to be selected for the individual patient [8]. Systemic inflammation is a recognised characteristic of malignancy [9] and a number of inflammatory markers have been investigated as prognostic indicators in cancer patients [10], [11].

One such biomarker, the neutrophil–lymphocyte ratio (NLR), has been associated with reduced survival in many solid tumours [12], [13]. As the differential white cell count is regularly performed in the management of cancer patients, NLR is a relatively simple and inexpensive biomarker to implement in routine clinical practice. However it is a usual practice to only record total white blood cell (WBC) count and absolute neutrophil count (ANC) in trial databases, which may restrict the wider use of NLR in the clinical trial setting. As a result a modified version, the derived NLR (dNLR), has been developed using WBC and ANC parameters and is reported to have a similar prognostic value to NLR, using an optimal cut-off value ≥ 2:1 [14].

In this study, we investigated the prognostic value of dNLR on progression-free survival (PFS) and overall survival (OS) in oesophageal cancer patients treated with dCRT in the SCOPE1 (Study of Chemoradiotherapy in OesoPhageal Cancer with or without Erbitux) trial. We also aimed to identify the optimal dNLR cut-off value in this patient group.

## Materials and methods

### Study design and setting

The primary objective of the randomised (1:1) phase 2/3 SCOPE1 study was to compare the effect of CRT with and without cetuximab on survival in patients with oesophageal cancer deemed unsuitable for surgery. 258 patients were recruited from 36 centres in the UK between February 2008 and January 2012. The CRT regimen consisted of 2 cycles of induction cisplatin–capecitabine chemotherapy followed by a further 2 cycles given concurrently with conformal external beam radiotherapy (50 Gy in 25 fractions over 5 weeks) with or without 12 weeks of cetuximab. The SCOPE1 adhered to the rules of CONSORT; the trial design, eligibility criteria and results have been reported previously [3], [15]. Written informed consent was obtained from all recruited patients. A blood sample was taken in the week prior to starting treatment in all patients enroled to the trial; WBC and ANC were documented on the case report form.

### Statistical methods

All statistical analyses were pre-planned and conducted using Stata SE 14. We calculated % of total dose (actual total dose divided by protocol total dose) and % dose intensity (actual dose intensity [dose per unit time] divided by protocol dose intensity) for each protocol drug as measures of compliance. As has been done elsewhere, patients who progressed or died during the treatment period had denominators calculated up to the point where they progressed or died [16]. Likewise for radiotherapy we calculated % of full protocol dose received by each patient and for those who progressed or died during the treatment period the denominator was calculated up to the point where they progressed or died.

A derived neutrophil to lymphocyte ratio was calculated using the formula [14]:

$$\text{dNLR}=\frac{\text{ANC}}{\text{WBC}-\text{ANC}}$$

dNLR was redefined as a binary variable by finding the value from a receiver-operating characteristic (ROC) curve that maximised the percentage correctly classified for predicting survival at 24 months (the median OS found in the first analysis of SCOPE1 [3]). The balance of this binary dNLR variable across prognostic characteristics of the SCOPE1 patients was assessed using chi square tests. Kaplan–Meier’s curves were used to display the prognostic value of the binary dNLR variable for different types of survival measure: overall survival (OS), progression free survival (PFS), local progression free survival inside the radiation volume (LPFSi), local progression free survival outside the radiation volume (LPFSo), and distant progression free survival (DPFS). We calculated survival from date of randomisation to when an event occurred i.e. progression or any death for PFS, and any death for overall survival. Patients who were event free were censored at the time they were last known to be event free. Univariable (the binary dNLR) and multivariable Cox regression models were used to assess the prognostic effect of dNLR on the different types of survival at two time points – pre-treatment (baseline) and following two cycles of induction chemotherapy prior to dCRT. The multivariable models included, in addition to the binary dNLR, SCOPE1 trial arm, age, reason for not receiving surgery, sex, WHO performance status at baseline, disease stage, tumour type, radiation compliance, cisplatin compliance, capecitabine compliance, and total disease length as covariates, and treating centre as a shared frailty. Additionally, a Cox model with a treatment–dNLR level interaction was used to assess whether the treatment effect differed between the two dNLR groups. In each case, the validity of the proportional hazards assumption was checked using Cox–Snell’s residuals and Schoenfeld’s global test.

## Results

Of the 258 patients recruited into the SCOPE1 trial between February 2008 and February 2012, 257 had both pre-treatment WBC and ANC results collected and were used in these analyses. The median follow-up (IQR) was 46.2 (35.9–48.3) months for surviving patients. The distribution of dNLR was positively skewed with a median of 1.86 (IQR: 1.46–2.43, range: 0.75–26.00). The ROC analysis of the sensitivity and specificity of dNLR in predicting death within 24 months after randomisation was performed on 250 (97.2%) patients (1 patient did not have a pre-treatment dNLR available and a further 7 were lost to follow-up prior to 24 months) (Supplemental material, Fig. S1). Using ROC curve analysis, the optimal dNLR cut-off value was calculated as 2.029 (sensitivity = 55.75%, specificity = 70.07%) (Supplemental material, Fig. S1).

Patient and tumour baseline characteristics according to dNLR (< 2 vs. ≥ 2) are shown in Table 1. Of the clinicopathological features analysed, sex, performance status, and total disease length were significantly associated with pre-treatment dNLR. However, if using a Bonferroni correction (*p* = 0.05/11 = 0.005), only sex was significantly associated with dNLR.

**Table 1:** Characteristics of the study participants, according to baseline dNLR level.

		dNLR < 2 (*n* = 146)		dNLR ≥ 2 (*n* = 111)		Test	
		*n*	%	*n*	%	Chi square	*p*
SCOPE1 trial arm	dCRT only	74	50.7	54	48.6	0.105	0.746
	dCRT + cetuximab	72	49.3	57	51.4		
Age	<65	65	44.5	44	39.6	0.615	0.433
	≥65	81	55.5	67	60.4		
Reason for not receiving surgery	Patient choice	59	40.4	38	34.2	1.413	0.493
	Local extent of disease	68	46.6	54	48.6		
	Comorbidity/poor PS	19	13.0	19	17.1		
Sex	Female	77	52.7	36	32.4	10.555	0.001
	Male	69	47.3	75	67.6		
WHO performance status	0	83	56.8	48	43.2	4.671	0.031
	1	63	43.2	63	56.8		
Stage	I or II	64	43.8	39	35.1	1.988	0.159
	III	82	56.2	72	64.9		
Tumour type	Squamous	111	76.0	77	69.4	1.423	0.233
	Adeno/undiff	35	24.0	34	30.6		
Full radiation dose	Yes	129	88.4	89	80.2	3.275	0.070
	No	17	11.6	22	19.8		
Cisplatin intensity	≥75%	107	73.3	75	66.6	0.998	0.318
	<75%	39	26.7	36	33.4		
Cape/5FU intensity	≥75%	103	70.6	70	63.1	1.606	0.205
	<75%	43	29.5	41	36.9		
Total disease length	<4 cm	30	20.5	26	23.4	8.672*	0.034
	≥4 to <6 cm	59	40.4	26	23.4		
	≥6 to <8 cm	26	17.8	29	26.1		
	≥8 cm	31	21.2	30	27.0		

(dNLR: derived neutrophil–lymphocyte ratio; dCRT: definitive chemoradiotherapy; WHO: World Health Organisation).

* Chi square test for trend.

**Fig. 1:**
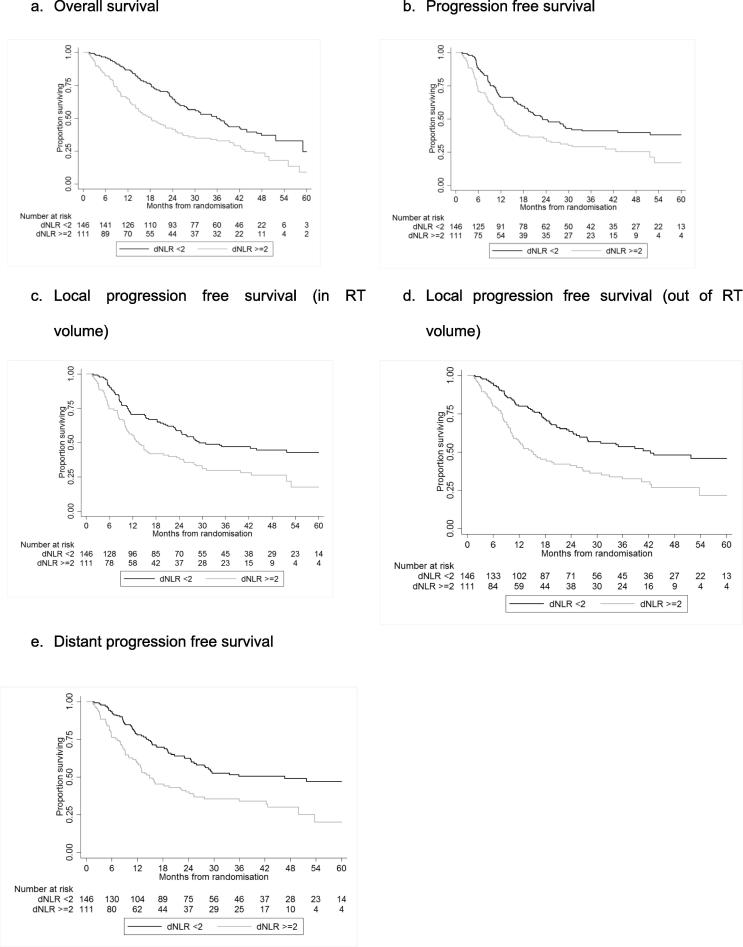
Kaplan–Meier’s curves of survival by baseline dNLR.

Kaplan–Meier’s curves according to the pre-treatment dNLR demonstrated a survival advantage for patients with dNLR < 2 (Fig. 1). Median OS was 36 months (95% CI 27.8–42.4) for patients with dNLR < 2 and 18.4 months (95% CI 14.1–24.9) for patients with dNLR ≥ 2. An elevated dNLR ≥ 2 was significantly associated with a decreased OS in both univariable analysis (HR 1.74 [95% CI 1.29–2.35], *p* < 0.001) and multivariable analysis (HR 1.64 [1.17–2.29], *p* = 0.004) (Table 2). In subgroup analysis, dNLR ≥ 2 was an independent prognostic factor for poor OS in patients with squamous cell carcinoma (HR = 2.06, 95% CIs: 1.25–3.41, *p* = 0.005) but the evidence was weaker for adenocarcinoma/undifferentiated tumours (HR = 2.52, 95% CIs: 0.88–7.23, *p* = 0.085) for whom the sample size was smaller (Supplemental material, Table S1). PFS, LPFSi, LPFSo and DPFS were all significantly reduced in patients with dNLR ≥ 2 in both univariable and multivariable analyses (Fig. 1; Supplemental material Table S1).

**Table 2:** Univariable and multivariable Cox regression analysis of overall survival.

Variable	Category	Time (months)			Univariable			Multivariable		
		*n*	Median	95% CIs	HR	95% CIs	*p*	HR	95% CIs	*p*
dNLR	dNLR < 2	146	36.0	27.8–42.4	1.00			1.00		
	dNLR ≥ 2	111	18.4	14.1–24.9	1.74	1.29–2.35	<0.001	1.64	1.17–2.29	0.004
Trial arm	CRT only	129	34.5	24.7–42.3	1			1.00		
	CRT + cetuximab	129	24.7	18.6–31.3	1.25	0.93–1.69	0.137	1.18	0.86–1.61	0.318
Age	<65	109	36.7	24.9–43.6	1			1.00		
	≥65	149	24.5	19.7–30.1	1.36	1.00–1.85	0.047	1.27	0.91–1.77	0.156
Reason no surgery	Patient choice	97	31.3	24.0–44.0	1			1.00		
	Local extent of disease	122	24.7	18.6–34.5	1.2	0.86–1.68	0.276	0.97	0.67–1.42	0.891
	Comorbidity/Poor PS	39	31.6	14.8–42.7	1.25	0.81–1.94	0.318	1.05	0.62–1.80	0.849
Sex	Female	113	34.6	24.7–48.8	1			1.00		
	Male	145	24.9	19.6–31.6	1.44	1.06–1.95	0.02	1.23	0.87–1.74	0.248
WHO status	0	131	30.3	24.0–38.4	1			1.00		
	1	127	24.9	19.2–34.3	1.14	0.84–1.53	0.405	0.95	0.67–1.35	0.776
Stage	I or II	103	42.4	31.3–49.9	1			1.00		
	III	155	24	18.6–26.8	1.65	1.20–2.27	0.002	1.51	1.04–2.20	0.031
Tumour type	Squamous	188	28.4	24.0–38.0	1			1.00		
	Adeno/undiff	70	24.9	15.9–35.1	1.24	0.90–1.72	0.192	1.02	0.68–1.54	0.926
Full radiation dose	Yes	217	34.3	25.8–39.1	1			1.00		
	No	41	10	5.9–18.4	3.19	2.17–4.70	<0.001	2.03	1.20–3.45	0.009
Cisplatin intensity	≥75%	182	35.9	27.2–42.4	1			1.00		
	<75%	76	16.2	12.5–20.8	2.18	1.59–2.99	<0.001	1.86	1.16–3.00	0.011
Cape/5FU intensity	≥75%	172	34.5	25.4–39.4	1			1.00		
	<75%	86	20	15.4–24.7	1.66	1.22–2.26	0.001	0.85	0.54–1.33	0.470
Total disease length	<4 cm	56	36	24.7–58.0	1			1.00		
	≥4 to <6 cm	85	37.9	24.0–49.9	0.98	0.63–1.52	0.928	1.07	0.67–1.71	0.768
	≥6 to <8 cm	55	24.9	18.6–40.3	1.46	0.92–2.33	0.107	1.13	0.67–1.92	0.638
	≥8 cm	62	18.4	14.9–27.8	1.84	1.17–2.89	0.009	1.57	0.94–2.62	0.083

**Fig. 2:**
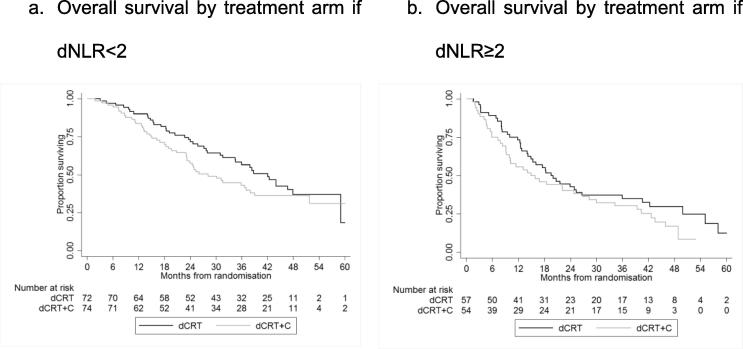
Kaplan–Meier’s curves of SCOPE1 treatment by dNLR.

In the analysis of OS according to arm allocation within the SCOPE1 trial, dNLR was not predictive for treatment effect (Fig. 2, Table 3). The addition of cetuximab to CRT was not associated with a survival difference for patients in either dNLR groups, with a *p*-value for interaction of 0.768.

**Table 3:** Effect of dNLR in predicting treatment effect.

		Time (months)			Univariable			Multivariable		
		*n*	Median	95% CIs	HR	95% CIs	*p*	HR	95% CIs	*p*
dNLR < 2	dCRT only	72	42.0	31.6–47.9	1.00					
	dCRT + cetuximab	74	28.2	23.2–38.0	1.26	0.82–1.92	0.290	1.23	0.77–1.96	0.382
dNLR ≥ 2	dCRT only	57	19.6	14.2–26.7	1.00					
	dCRT + cetuximab	54	15.9	9.26–26.8	1.37	0.88–2.12	0.159	1.24	0.67–2.28	0.494

(dNLR: derived neutrophil–lymphocyte ratio; dCRT: definitive chemoradiotherapy).

We repeated the multivariable analyses in a sensitivity analysis that excluded patients who died during treatment (6 patients) and the findings above were unchanged.

The prognostic value of a repeat dNLR following 2 cycles of induction chemotherapy but prior to the commencement of dCRT was also analysed. Of the 258 patients, 7 were lost to follow up prior to 2 years and 36 had missing pre-CRT WBC or ANC results, leaving 215 in the analysis. The distribution of dNLR at this time point was positively skewed with a median of 1.19 (IQR: 0.85–1.59, range: 0.33–4.86). Using ROC curve analysis, the optimal dNLR cut-off value for predicting 2 years of survival was calculated as 1.5 (correctly classified 60.5%) (Supplemental material, Fig. S2). An elevated dNLR ≥ 1.5 was significantly associated with a decreased OS in univariable analysis (HR 1.56 [95% CI 1.10–2.21], *p* = 0.014) but not in multivariable analysis (HR 1.45 [0.99–2.12], *p* = 0.054).

## Discussion

In this present study, an elevated pre-treatment dNLR was an independent prognostic biomarker for OS and PFS in oesophageal cancer patients treated with definitive CRT. dNLR was also significantly associated with OS outcomes in patients with squamous cell carcinoma, and to a lesser extent in adenocarcinoma/undifferentiated tumours. Measurement of dNLR following two cycles of induction chemotherapy (but prior to commencement of dCRT) was not associated with OS outcomes in multivariable analysis.

Inflammation in the malignant setting is known to promote both tumorigenesis and disease progression, and reduces response to systemic treatments [17]. Proctor et al. [14] originally validated dNLR as an alternative to NLR in a retrospective cohort study of more than 12,000 cancer patients with a variety of malignancies, including oesophageal tumours. Baseline dNLR ≥ 2 was associated with worse overall survival compared to a dNLR < 2 (HR 1.76; *p* < 0.001); cancer-specific survival was also reduced (HR 1.83; *p* < 0.001) [14]. In the present study, we calculated the optimal dNLR cut-off value to be 2, externally validating this threshold level.

The prognostic value of dNLR has since been investigated in several malignancies including breast [18], urological [19], colorectal [20], and upper gastrointestinal tumours [21], [22], [23]. However to our knowledge, our study is the first to investigate the prognostic role of dNLR in oesophageal cancer.

There is evidence for the use of NLR as a prognostic indicator of survival in oesophageal cancer. A meta-analysis of over 1500 patients from seven retrospective cohort studies confirmed that an elevated pre-treatment NLR was prognostic for OS but not disease-free survival [24]. T3–4 disease and lymph node disease were also significantly associated with NLR. However the study included pooled data from largely Asian, retrospective surgical series which included the use of both neoadjuvant and adjuvant CRT, resulting in significant between-study heterogeneity. Furthermore, the studies included employed a variety of NLR cut-off values ranging between two and five.

The only study to evaluate NLR in oesophageal cancer patients treated with definitive CRT confirmed that a raised NLR was an independent prognostic factor for both OS and PFS in 138 patients [25]. The patient population was similar to that of our study, the majority having locally-advanced, predominantly inoperable squamous cell carcinoma. However it was a retrospective study, there were significant differences in the chemotherapy regimens used and radiotherapy included treating both the primary and involved nodes up to a total dose of 63 Gy with considerably larger target volume margins and regional elective nodal irradiation. Furthermore, the median OS for all patients was significantly lower than that of the SCOPE1 trial, reported at only 19.9 months (range 1.1–97.2) [25]. Despite these differences in regimens and OS between cohorts, NLR proved to be discriminatory in both the studies, and would suggest that this is a useful baseline variable for assessing prognosis in oesophageal cancer patients being considered for dCRT.

The major implication of dNLR would be to risk-stratify patients, assisting the clinician and patient to make an informed decision about treatment options. For example, in patients who are borderline fit for dCRT, it is often difficult to quantify potential benefits of a given treatment versus the real risk of significant and potentially life-threatening toxicities. Discussions using pre-treatment dNLR results may assist the individual patient to weigh up whether the side-effects of dCRT are worth risking, particularly if they are considered to have a poor prognosis as determined by a raised pre-treatment dNLR, or whether they would prefer to employ a more conservative treatment strategy. Given the variation in survival, consideration should also be given to use dNLR as a stratification factor for future dCRT trials.

The strengths of this current study is that the data were prospectively collected, in the setting of a randomised controlled trial which has now standardised treatment of oesophageal cancer with dCRT in the UK. dNLR was available for all patients within one week of starting treatment in the patients included in our analysis. However, the dNLR cut-off value found in this study will need to be validated in further independent datasets. Whilst this study has validated pre-treatment dNLR as a potential prognostic biomarker for oesophageal cancer patients treated with dCRT, analysis of other haematological components, albumin and other markers of the systemic inflammatory response such as C-reactive protein (CRP) was not conducted.

In conclusion, we have demonstrated that an elevated pre-treatment dNLR is a potential independent prognostic marker for both OS and PFS in oesophageal cancer treated with dCRT. It serves as a readily available tool for risk-stratifying patients and should be considered as a stratification factor in future clinical trials aiming to optimise non-surgical treatment strategies for locally-advanced oesophageal cancer.

## Conflict of interest statement

Samantha Cox has received educational sponsorship from Merck Serono and Pfizer to attend an international conference.

All remaining authors have declared no conflicts of interest.
