# Supplementary figures and images for: The prognostic value of derived neutrophil to lymphocyte ratio in oesophageal cancer treated with definitive chemoradiotherapy
Source: Radiother Oncol. 2017 Oct;125(1):154–9. doi: 10.1016/j.radonc.2017.08.023 (PMC5648078; doi:10.1016/j.radonc.2017.08.023)

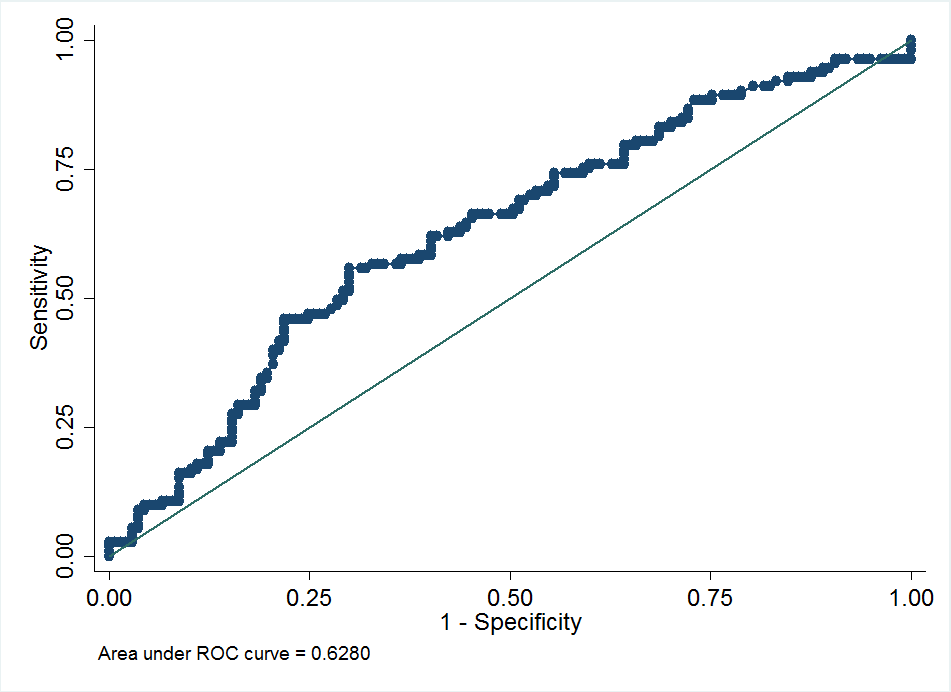

Supplement: Supplementary Fig. S1 [file mmc1.doc]

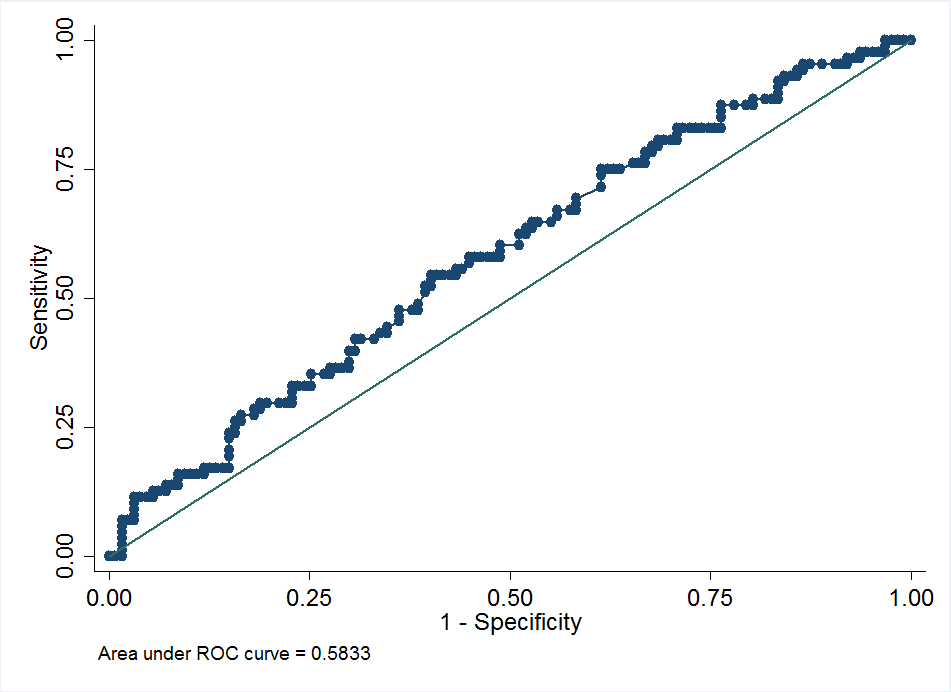

Supplement: Supplementary Fig. S2 [file mmc2.doc]
