# Supplementary material for: The prognostic value of derived neutrophil to lymphocyte ratio in oesophageal cancer treated with definitive chemoradiotherapy
Source: Radiother Oncol. 2017 Oct;125(1):154–9. doi: 10.1016/j.radonc.2017.08.023 (PMC5648078; doi:10.1016/j.radonc.2017.08.023)
Supplement: Supplementary Table S1 [file mmc3.doc]

|  |  | Time (months) | | | Univariable | | | Multivariable | | |
| --- | --- | --- | --- | --- | --- | --- | --- | --- | --- | --- |
|  |  | **n** | **Median** | **95% CIs** | **HR** | **95% CIs** | **p** | **HR** | **95% CIs** | **p** |
| OS in squamous | dNLR <2 | 111 | 38.0 | 28.2-59.0 | 1.00 |  |  | 1.00 |  |  |
|  | dNLR ≥2 | 77 | 19.6 | 14.9-25.4 | 1.95 | 1.36-2.81 | <0.001 | 2.06 | 1.25-3.41 | 0.005 |
| OS in adeno/undiff | dNLR <2 | 35 | 27.2 | 20.9-38.4 | 1.00 |  |  | 1.00 |  |  |
|  | dNLR ≥2 | 34 | 12.8 | 8.4-42.7 | 1.31 | 0.75-2.26 | 0.340 | 2.52 | 0.88-7.23 | 0.085 |
| PFS | dNLR <2 | 146 | 23.2 | 18.4-33.7 | 1.00 |  |  | 1.00 |  |  |
|  | dNLR ≥2 | 111 | 12.4 | 10.0-15.3 | 1.68 | 1.23-2.29 | 0.001 | 1.48 | 1.05-2.07 | 0.025 |
| LPFSi | dNLR <2 | 146 | 29.1 | 23.2-60.6 | 1.00 |  |  | 1.00 |  |  |
|  | dNLR ≥2 | 111 | 13.6 | 10.4-21.4 | 1.84 | 1.34-2.54 | <0.001 | 1.70 | 1.19-2.42 | 0.003 |
| LPFSo | dNLR <2 | 146 | 42.4 | 27.8-∞ | 1.00 |  |  | 1.00 |  |  |
|  | dNLR ≥2 | 111 | 15.3 | 10.7-25.4 | 2.07 | 1.47-2.91 | <0.001 | 1.87 | 1.24-2.82 | 0.003 |
| DPFS | dNLR <2 | 146 | 46.6 | 25.9-∞ | 1.00 |  |  | 1.00 |  |  |
|  | dNLR ≥2 | 111 | 14.9 | 11.8-23.5 | 1.95 | 1.40-2.74 | <0.001 | 1.75 | 1.16-2.62 | 0.007 |
